# Telehealth: A Resource for Vulnerable Populations to Access Oral Healthcare During the COVID-19 Pandemic

**DOI:** 10.1093/geroni/igab046.1804

**Published:** 2021-12-17

**Authors:** Victoria Raveis, David Glotzer, Andre Ritter

**Affiliations:** New York University, New York, United States

## Abstract

Dental care and adherence to daily oral hygiene practices are particularly important for healthy aging. For socially disadvantaged or impoverished, older community residents, populations who are at risk for long-standing oral disease, public dental clinics are safety nets. In March 2020, when COVID-19 cases surged in the New York metropolitan area, a months-long suspension of the area’s community dental services occurred, including clinical operations at the NYU College of Dentistry. To ameliorate the impact of this widespread service suspension, NYU Dentistry implemented an interim Dental Telehealth Service, open to the community, with telehealth consultations delivered by NYU faculty. This consultative service served a diverse population, adhering to guidelines the American Dental Association (ADA) issued on “urgent” and “emergency” care, with the goal of treating with a minimally invasive approach, to relieve the burden on hospital emergency rooms. Older adults, experiencing dental issues and fearing they were particularly vulnerable to the virus, called into this service, as they desperately wanted to avoid the overwhelmed public hospital ERs. A range of significant dental issues, i.e. pain, swelling, tooth fractures, were addressed. Implementing this community resource was a creative strategy to address a serious health services gap during this public health crisis. It also yielded important insights regarding the feasibility, acceptability and utility of telehealth, as a routine component of dental practice, when treating older adults, who often have serious co-morbidities and limited mobility. Certainly, the conversational nature of telehealth is a less stressful and anxiety-provoking clinical encounter.

